# Methylglyoxal impairs human dermal fibroblast survival and migration by altering *RAGE-hTERT* mRNA expression *in vitro*

**DOI:** 10.1016/j.toxrep.2024.101835

**Published:** 2024-11-26

**Authors:** Nurul Muhammad Prakoso, Ayu Mulia Sundari, Fadhilah, Abinawanto, Anantya Pustimbara, Astari Dwiranti, Anom Bowolaksono

**Affiliations:** a Master Program in Biology, Department of Biology, Faculty of Mathematics and Natural Science, University of Indonesia, Depok, West Java 16424, Indonesia; b Cellular and Molecular Mechanisms in Biological System (CEMBIOS) Research Group, Department of Biology, Faculty of Mathematics and Natural Science, University of Indonesia, Depok, West Java 16424, Indonesia; c Tokyo Institute of Technology, School of Life Science and Technology, 4259 Midori-ku, Yokohama 2268501, Japan; d Center for Photodynamic Medicine, Kochi Medical School, Kohasu, Oko-cho, Nankoku-shi, Kochi 783-8505, Japan

**Keywords:** Human dermal fibroblasts, Methylglyoxal, Reactive oxygen species, Dermal toxicity

## Abstract

Fibroblasts are native residents in dermal layer of human skin which are important for dermal regeneration and essential during cutaneous wound healing by releasing inflammatory markers and actively migrate to close an open wound. Premature skin ageing due to methylglyoxal (MGO) has recently caught the attention considering its potential to accelerate the emergence of skin ageing signs, however previous studies were only focused in primary neonatal dermal fibroblast and NIH3t3 fibroblast cell line. Therefore, thorough investigation is required to study the impact of MGO on primary human dermal fibroblast isolated from adult subject (HDFa). In our experiments, short exposure of MGO was observed to induced significant reductions in cell viability at concentrations of 7.5, 10, 12.5, 15, and 17.5 mM (*p* < 0.005) after 3 hours of treatment. The cellular death of HDFa at 10, 12.5 and 15 mM of MGO were also marked by increased in intracellular ROS level, indicating the involvement of oxidative stress-induced death in these cells. We also observed enlarge scratch areas of cells exposed with 7.5 and 10 mM MGO compared to control after 26 hours, thereby suggesting a decline in cell migration and viability in this group. We propose the increased ROS as the consequence of AGE-RAGE activation which was marked by significant elevation of *RAGE* mRNA on cells exposed to 10 mM MGO. Our data also suggest the occurrence of DNA damage events via ROS-induced oxidation or mediated by decline in *hTERT* mRNA expression.

## Highlights

• MGO causes cellular toxicity on primary adult human dermal fibroblast.
• Impaired cell migration was argued to be related to increased ROS as a result of the activation of AGE-RAGE axis.
• Observed DNA damage events may be exacerbated by decreased hTERT mRNA expression.

## Graphical Abstract

**Figure:**
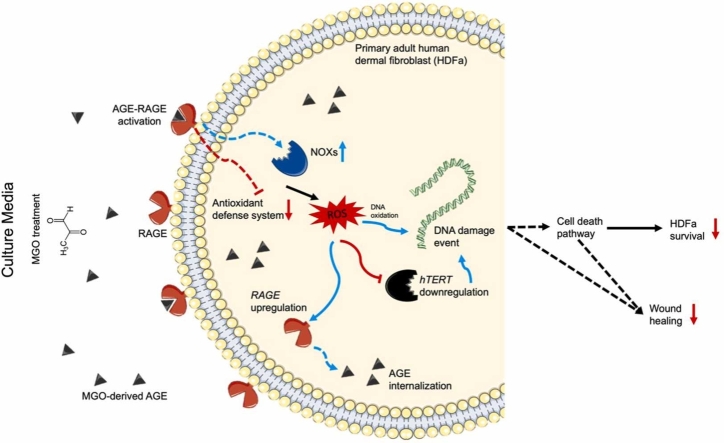

## 1 Introduction

Viability of fibroblasts residing in human dermis and their state of metabolism are indispensable parts of maintaining a health and youthful skin [1], [2]. The presence of metabolically active dermal fibroblast is not only essential in supporting regeneration of dermal layer, but also massively important during the rejuvenation of extracellular matrix (ECM) by synthesizing structural proteins [3]. Balanced processes of production and degradation of ECM provides a stable living vessel for other skin structure and directly contributes to skin appearance by retaining contour [4]. The role of dermal fibroblast during wound healing is irreplaceable since their migration and proliferation promote wound closure by expressing collagen type I and III and recruit immune cells by releasing inflammatory cytokines [5], [6]. However, the amount of actively dividing fibroblast and its metabolism have been reported to decline due to intrinsic ageing, according to plenty of research. In a study published by Solé-Boldo et al. in 2020, they reported a reduction in fibroblast count biopsied from old subject compared to younger group. In the same RNA-seq analysis, they also mentioned delayed cell cycle transition from G1-S phase in old fibroblast [7]. These findings were in agreement with reports from other studies, in which intrinsic ageing has been shown not only to weaken fibroblast proliferation, but also impairs its clonogenic ability and delays migration both in 2D and 3D culture conditions [2], [8], [9], [10].

Unfortunately, the presence of aged fibroblast in dermal layer of human skin may be accelerated due to external exposure, such as accumulation of advanced glycation end-products (AGE) which has drawn a significant attention as an emerging factor of skin ageing in recent years [11]. In other types of cells, excessive build-up of AGE is the result of an elevated glycation on cellular biomolecules such as proteins, lipids, and nucleic acids, which may interfere their function or disturb mechanical contact with their substrates [12]. Exogenous AGE molecules may enter the cells via binding to receptor of advanced glycation end-products (RAGE) [13]. In addition to taken exogenously, a precursor of AGE is also synthesized by cells via glycolysis pathway by forming a by-product called methylglyoxal (MGO) [14]. MGO is classified as a highly reactive dicarbonyl species which react to proteins and DNA to generate advanced glycation end-products (AGE) [15]. The cellular concentration of MGO could be elevated with excessive sugar consumption, such as glucose and fructose, by increasing the activity of glycolysis [16]. A constant production of AGE due to MGO leads to elevated expression of RAGE which mediates the transport of AGE to enter the intracellular compartment, and subsequently activates proinflammatory markers, generating reactive oxygen species (ROS), and impairs antioxidant defence system [11], [15]. In this situation, DNA damage events are thought to be inevitable as a result of oxidative stress. The highly reactive MGO also interact with guanine, which may lead to DNA strand breaks and replication error due to the formation of MGO-DNA adducts [17].

In dermal layer of human skin, the accumulation of AGE had a negative impact on the stability of ECM as it causes crosslinking of biomolecules with long half-lives such as collagen type I and IV, which ultimately leads to reduced skin flexibility due to impairment in collagen regeneration [18]. Although many reports have described the elevated AGE levels in skin, its quantification was only done with skin autofluorescence measurement, therefore the data could not be expressed in concentration [19], [20]. Meanwhile in blood circulation, the concentration of MGO was reported to be between 50—300 nM and was found to increased to almost 3-folds in diabetic patients [21], [22]. Interestingly, the *in vivo* concentrations of MGO were distinctly reported between tissue samples. It has been reported that the human cataractous lens contains 1—2 µM of MGO, while higher concentrations were required to induced a significant cellular toxicity in human primary astrocytes (3.39 mM) and Rat islet tumour cell line RINm5F (10 mM) which was used to demonstrate the polyneuropathy and pancreatic cell deaths in patients with diabetes [23], [24], [25].

Toxicity studies of MGO which reported a reduction in survival and elevated level of cell senescence were only performed in neonatal skin fibroblast [26] and NIH3t3 fibroblast cell line [27], providing limited evidence on its impact on adult dermal fibroblast. Moreover, experimental data regarding the role of MGO accumulation in impaired migration during wound healing of dermal fibroblasts also requires experimental evidence. Therefore, to our knowledge, the experimental data of MGO exposure on primary adult human dermal fibroblast (HDFa) is urgently required to better understand MGO-induced ageing process of human skin. The objective of this study was to investigate the pathological mechanism of MGO on HDFa and the involvement of *RAGE*-*hTERT* expression in relation to reduced survival and DNA damage events. We also questioned migration ability of MGO-induced HDFa considering their vital role during wound closure.

## 2 Materials and methods

### 2.1 Materials

Primary adult human dermal fibroblast (HDFa) was purchased from Hayandra Laboratory which was isolated from the foreskin of normal adult. Methylglyoxal 40 % (Cat. No. M0252), Dulbecco’s Modified Eagle’s Medium (DMEM) with high-glucose (Cat. No. D6429), Fetal Bovine Serum (Cat. No. F2442), Antibiotic-Antimycotic Solution 100x (Cat. No. A5955), and Accutase solution (Cat. No. A6964) were purchased from Sigma-Aldrich, Darmstadt, Germany. The MTS CellTiter 96-Aqueous Non-Radioactive Cell Proliferation Assay (Cat. No. G5421) was purchased from Promega, Wisconsin, USA. Reactive Oxygen Species (ROS) Detection Assay Kit was purchased from Abcam, Boston, Massachusetts, USA. Blood/Cell Total RNA Mini Kit (RB100) and Genomic DNA Mini Kit (Blood/Cultured Cell) (Cat. No. GB300) were purchased from Geneaid Biotech Ltd., New Taipei, Taiwan. The ReverTra-Ace™ qPCR RT Master Mix with gDNA Remover (Cat. No. FSQ-301) and KOD SYBR® qPCR Mix (Cat. No. QKD-201) were purchased from Toyobo, Co. Ltd., Shanghai, China.

### 2.2 Ethical approval

This research was conducted at the Integrated Cellular and Molecular Biology Laboratory, Department of Biology, Faculty of Mathematics and Natural Science, University of Indonesia. The ethical exemption letter (KET-451/UN2-F1/ETIK/PPM.00.02/2024) was given by The Health Research Ethics Committee, Faculty of Medicine, University of Indonesia-Cipto Mangungkusumo Hospital before conducting experiments.

### 2.3 Cell culture

The primary human dermal fibroblast (HDFa) was cultured in high-glucose DMEM supplemented with 10 % FBS and 1x antibiotic-antimycotic solution. To support cell proliferation, the culture was grown in a humidified environment with 5 % CO_2_ at 37°C until 80 % confluence [28]. The culture maintenance was performed by passaging the cells prior to experiments. Only cells with passages 5—9 were used to collect experimental data.

### 2.4 Measurement of cell survival

The survival of HDFa was determined by 3-(4,5-dimethylthiazol-2-yl)-5-(3-carboxymethoxyphenyl)-2-(4-sulfophenyl)-2H-tetrazolium (MTS) assay according to the manufacturer’s protocol [29]. Initially, 10,000 cells were seeded into a clear 96-well plate and incubated for approximately 24 hours to allow attachment. The culture media were replaced with fresh media containing MGO at different concentrations (5, 7.5, 10, 12.5, 15, and 17.5 mM) or only fresh complete media as control. The cells were incubated for 3 hours and washed with Dulbecco’s phosphate buffered saline (DPBS) to remove traces of MGO in the culture. The mixture of MTS and fresh media was transferred to each well and incubated for 3 hours to allow the conversion of soluble formazan. The survival of HDFa was quantified by spectrophotometric measurement at 490 nm using Varioskan™ Lux Multimode Microplate Reader.

### 2.5 Quantification of intracellular reactive oxygen species (ROS)

The release of intracellular ROS was quantified based on H_2_DCFDA fluorometric assay following manufacturer’s instruction for adherent cells [30]. Briefly, 10,000 cells were loaded into a black 96-well plate and left overnight. The cells were washed with 100 µL ROS assay buffer before being treated with fresh media containing 10, 12.5, and 15 mM of MGO or only fresh complete media as control. After three hours of incubation, another washing step was performed before adding of 100 µL of 1x ROS label into the cells. The cells were incubated for 45 minutes to allow the oxidation of H_2_DCF by intracellular ROS generated by MGO treatment. The amount of ROS was quantified by fluorometric readings at Ex. 495/Em. 529 nm using Varioskan™ Lux Multimode Microplate Reader. The fluorescence intensity was normalized against the absorbance of formazan readings at 490 nm to account for cell density differences. The presence of H_2_DCF-labeled cells was also observed using Nikon ECLIPSE Ni-L fluorescence microscope.

### 2.6 Scratch assay

The capacity to migrate was observed by scratch method in a 6-well plate. Fibroblast were seeded at cell density of 300,000 cells/well and incubated until confluence. A scratch was made using a 1000 µL micropipette tip in the middle of each well to simulate the formation of open wound. The cells were incubated with fresh media containing 7.5 and 10 mM of MGO or fresh complete media as control for three hours, and then washed with DPBS. A fresh culture media was added into each well. The migration and survival of HDFa was observed after incubated for 26 hours [31].

### 2.7 Gene expression analysis

HDFa culture was grown at 500,000 cells/well in a 6-well plate. The culture was left overnight to allow a proper adhesion of HDFa to the plate. The treatment was performed by incubation with fresh media containing 5, 7.5, and 10 mM of MGO or only fresh complete media as control for three hours. After incubation, HDFa were removed from the culture plate and washed with DPBS prior to cell lysis with RB buffer and β-mercaptoethanol. Ethanol was added to facilitate RNA precipitation and bind the RNA to the column matrix. Impurities were removed by washing multiple times before commencing DNase digestion to collect the final total RNA. Synthesis of complementary DNA (cDNA) was performed with following conditions: DNase I treatment at 37°C for 5 minutes, followed by reverse transcription at 37°C for 15 minutes, 50°C for 5 minutes, and 98°C for 5 minutes. The quantification of gene expression was performed on hTERT and RAGE [Table 1] using Qiagen Rotor-Gene Q Real-Time PCR System as follows: pre-denaturation at 98°C for 2 minutes, denaturation at 98°C for 10 seconds, annealing at 60°C for 10 seconds, and extension at 68°C for 35 seconds. The fold-change of hTERT and RAGE were calculated against GAPDH as reference.

**Table 1:** Primer sequences for gene expression analysis.

Gene	Name	Sequence (5’ → 3’)	Reference
GAPDH	GAPDH-F	CCACCCATGGCAAATTCC	[32]
	GAPDH-R	CAGGAGGCATTGCTGATGAT	
RAGE	RAGE-F	AACCAGGCGAGGAGGGGCCAACT	[33]
	RAGE-R	CACGCTCCTCCTCTTCCTCTGGTTTTCTG	
hTERT	hTERT-F	CCCATTTCATCAGCAAGTTTGG	[32]
	hTERT-R	CTTGGCTTTCAGGATGGAGTAG	

### 2.8 Observation of DNA damage events

Genomic DNA from each group were extracted by incubating previously detached HDFa with GB buffer at 60°C for 10 minutes. Absolute ethanol was transferred to the solution to allow DNA to bind to the column matrix. Multiple purification with wash buffer were performed to remove other cellular components. The level of DNA damage was measured by observing the presence of DNA smearing pattern in 0.8 % agarose gel electrophoresis.

### 2.9 Statistical analysis

Statistical analysis and data visualization were conducted using GraphPad Prism 10. One-way ANOVA statistical analysis was performed on cytotoxicity and ROS assay to calculate the significant difference between treatments and control. Unpaired t-test was employed to analyse the significant difference of gene expression after calculating 2^-ΔΔCt^ of each qPCR data. Statistical significance was defined as a p < 0.05. All data in the figure are presented in mean values ± standard deviation. The number of independent replications was displayed in each figure legend.

## 3 Results

### 3.1 Short exposure of MGO reduced HDFa survival

Previous clinical findings revealed a significant reduction in dermal thickness in the abdominal skin of diabetic patients compared to healthy individuals which was believed to be caused by AGE accumulation [34]. However, different research has examined distinct cellular consequences were induced by each AGE precursor on keratinocytes cell line [35]. Therefore, we initiated this study by investigating the potential toxicity of MGO with MTS assay to measure the survival of HDFa.

**Fig. 1:**
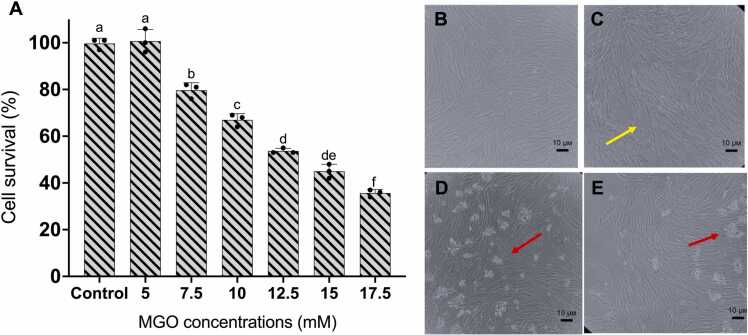
Measurement of HDFa survival using MTS assay. Toxicity of MGO on HDFa was increased in a dose-dependent manner after 3 hours (A). Normal cell shape and cell-to-cell contact of HDFa (B). HDFa treated with 7.5 mM MGO showing distinct cell shape as shown by the yellow arrow (C). The presence of detached cells in HDFa treated with 10 mM (D) and 12.5 mM (E) MGO as shown by the red arrows. One-way ANOVA was performed to determine statistical significance. Quantitative data are expressed as mean values with ±SD represented by error bars; n = 3, p < 0.05.

Exposure to 7.5 mM MGO reduced the survival significantly by almost 25 %. A significant reduction was also noted with increasing doses of MGO at 10, 12.5, 15, and 17.5 mM which indicated that the exposure of MGO may activated cellular death mechanism in HDFa. Brightfield microscopic observations were in agreement with MTS colorimetric measurement. We monitored distinct morphological feature of HDFa treated with 7.5 mM MGO, as disrupted cell shape and the loss of cell-to-cell contact were observed. Moreover, we also marked a noticeable number of detached cells in HDFa treated with 10 and 12.5 mM MGO (Fig. 1). This quantitative and observational analysis provided supporting evidence that reduced HDFa survival and proliferation might massively contribute to dermal toxicity of MGO, thereby reducing the dermal thickness of aged skin.

### 3.2 MGO promoted ROS-induced cell death in HDFa

In previous report, the exposure of AGE precursor has been reported to induce a significant increase in intracellular and mitochondrial superoxide production in HaCaT cells, in which cells treated with MGO was not only generated the highest level of intracellular and mitochondrial superoxide, but also increased the expression of SOD1 and SOD2 mRNA [35]. In this study, we quantified the level of ROS generation by employing H_2_DCFDA-based assay which covered the detection of hydroxyl, peroxyl, and other class of ROS in live cells [36].

**Fig. 2:**
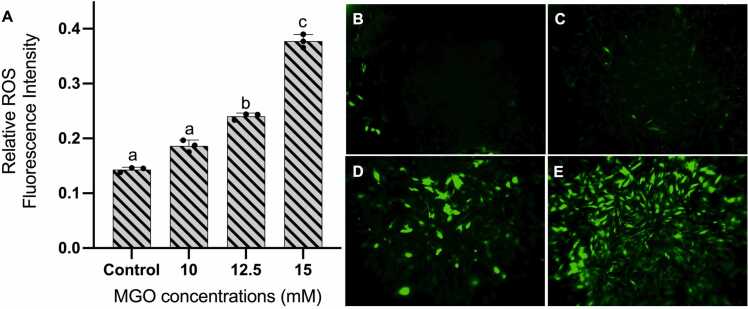
Quantitation of intracellular ROS using H_2_DCFDA assay in live cells. Elevated ROS production in HDFa treated with 10, 12.5, and 15 mM MGO (A). The fluorescence intensity was normalized against the absorbance of formazan at 490 nm to minimize the variable of cell count. Observation of ROS-positive HDFa using fluorescence imaging confirmed increasing ROS-positive cells from control (B) to cells treated with 10 mM (C), 12.5 mM (D), and 15 mM (E) MGO for 3 hours. One-way ANOVA was performed to determine statistical significance. Quantitative data are expressed as mean values with ± SD represented by error bars; n = 3, p < 0.05.

In quantitative measurement with microplate reader, there were increased ROS production in cells treated with 10, 12.5, and 15 mM MGO. This elevated ROS production was observed to be dose-dependent and was statistically significant after treated with 15 mM MGO. Moreover, visual observation using fluorescence microscope confirmed considerable increase in ROS-positive cells, in which cells treated with 15 mM demonstrating the highest number of ROS-positive (Fig. 2). Therefore, this data would suggest that increased ROS production might contribute to reduction in HDFa survival during MGO exposure.

### 3.3 MGO impairs wound healing in HDFa by inhibiting migration and survival

Increasing sugar consumption, such as glucose and fructose, have been reported to elevate MGO concentration with and without the presence of insulin in primary rat hepatocyte [16]. In another report, human foreskin fibroblast treated with high glucose demonstrated reduction in cell migration, therefore explaining the impaired wound healing due to decline in migration ability. However, the production of methylglyoxal (MGO) as a result of glucose metabolism was not investigated in the previous report [37], thereby created a missing link in the pathogenesis of reduced wound healing due to MGO.

**Fig. 3:**
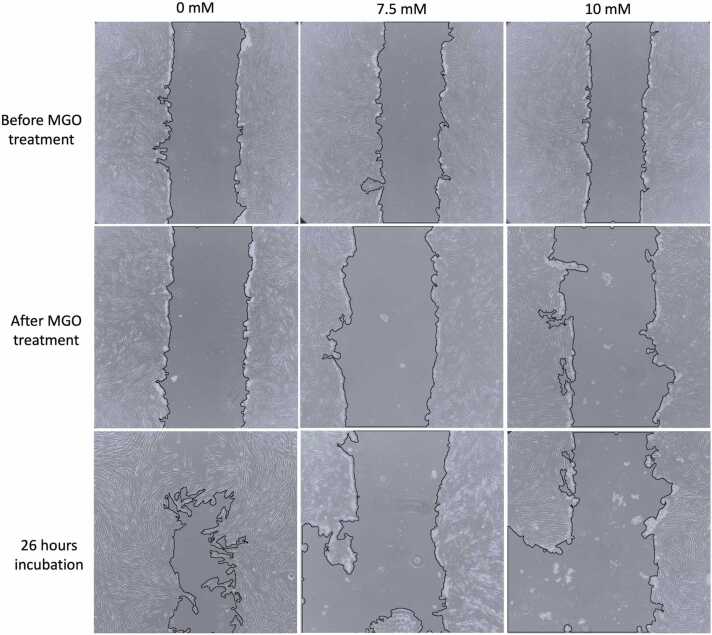
Visual inspection of HDFa migration ability exposed to 0, 7.5, and 10 mM MGO for 3 hours. Note reduced survival and impaired migration as enlarged scratch area in cells treated with 7.5 and 10 mM MGO were observed compared to control.

In this study, we investigated MGO as the potential mediator on impaired migration by employing scratch assay in a 6-well plate to observe wound closure activity of HDFa. In control group, we noted a reduction in wound gap after 26 hours of incubation which demonstrate typical migration of HDFa during wound closure. However, in treatment groups, we observed increased in scratch areas after being treated with 7.5 and 10 mM MGO for 3 hours. These group of cells did not show similar wound closure response even after incubated for 26 hours, indicating the lack of migration (Table 2). In addition to impaired cell migration, our data also suggest decline in survival of cells exposed to 7.5 and 10 mM MGO which contributed significantly to impaired wound closure as enlarged scratch areas were observed in Fig. 3.

**Table 2:** Quantitative measurement of HDFa migration under the exposure of 7.5 and 10 mM MGO for 3 hours.

	Before MGO treatment		After MGO treatment		26 hours incubation	
	Area (pixels^2^)	Scratch area (%)	Area (pixels^2^)	Scratch area (%)	Area (pixels^2^)	Scratch area (%)
0 mM	69,976 ± 11.22	33.65	76,009 ± 6.995	38.050	33,608 ± 27.69	16.163
7.5 mM	62,234 ± 11.91	30.061	88,445 ± 21.68	42.722	106,737 ± 55.38	51.333
10 mM	56,663 ± 8.08	27.251	91,023 ± 27.70	43.775	110,499 ± 68.07	53.141

### 3.4 MGO induced DNA damage and altered the expression of AGE receptor and human telomerase

**Fig. 4:**
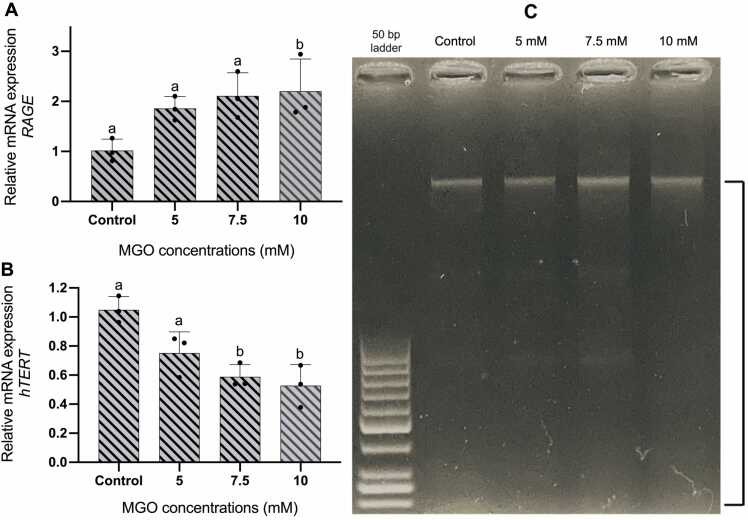
Gene expression analysis and visualization of potential DNA damage. Increased mRNA expression of RAGE due to MGO in a dose-dependent manner (A). Exposure to MGO significantly reduced hTERT mRNA expression in 7.5 and 10 mM MGO after 3 hours (B). Visualization of agarose gel electrophoresis showing higher degree of DNA smearing in cells treated with 7.5 and 10 mM MGO (C). Note higher degrees of DNA smearing pattern shown in black lines area. Unpaired t-test was performed to determine statistical significance. Quantitative data are expressed as mean values with ±SD represented by error bars; n = 3, p < 0.05.

Higher ROS concentration in cells exposed to MGO was previously described to be mediated by activation of RAGE upon binding with AGE, this triggers intracellular ROS production via NADPH oxidase (NOX) pathway and impairs antioxidant defence mechanism [11]. We then investigated the mRNA expression of RAGE to understand whether elevated ROS level in HDFa was accompanied by higher RAGE expression. Our qPCR result showed increases of RAGE mRNA expression in HDFa exposed to 5 and 7.5 mM MGO for 3 hours, while a significant increase in cells treated with 10 mM MGO was noted (Fig. 4).

Elevation of intracellular ROS poses a significant risk of DNA damage which could be fatal for cell survival. Moreover, MGO has been reported to react with DNA to form MGO-DNA adduct which could lead to DNA strand breaks and disrupt DNA replication [17]. We verified for a possible DNA damage by running 300 ng of extracted DNA on 0.8 % agarose electrophoresis and identified visually higher level of smearing which could indicate DNA damage events in HDFa exposed to 7.5 and 10 mM MGO (Fig. 4). Our qPCR data also revealed a significant decline in hTERT expression in cells treated with 7.5 and 10 mM MGO which could eventually exacerbate DNA damage due to failure of human telomerase in retaining DNA strands.

## 4 Discussion

**Fig. 5:**
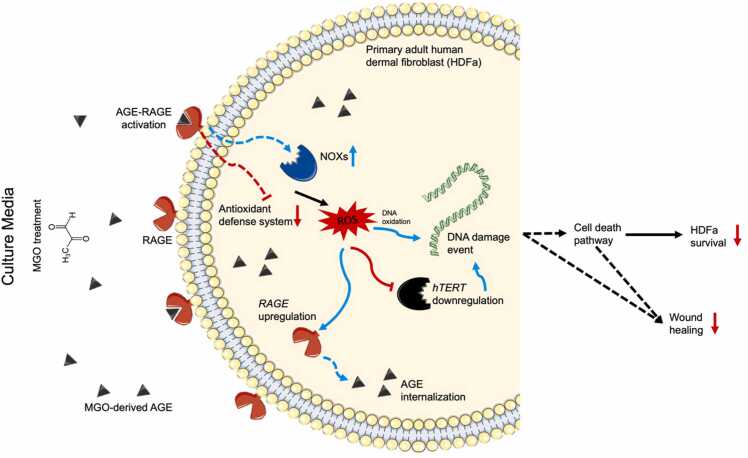
Schematic diagram of cellular and molecular mechanism of MGO toxicity on HDFa.

In accordance with report in HaCaT cell line, our data also suggest the toxic effect of MGO on primary adult human dermal fibroblast (HDFa) which we convinced was related to the increased in intracellular ROS concentration. This agreement would suggest similar physiological responses in HDFa and HaCaT (Fig. 5), which showed the presence of MGO in cells amplified the level of AGE which could interact with receptor of advanced glycation end-product (RAGE), thereby causing cellular oxidative stress by activating NADPH oxidases and limiting the expression of antioxidant genes, such as SOD1, SOD2, and CAT [35]. This evidence is supported by our data in which the expression of RAGE mRNA was increased in cells with the highest ROS-positive cells. We also argue the impaired HDFa migration was also affected by increased in ROS production mediated by AGE-RAGE activation, since exposure to high glucose was previously shown to delayed fibroblast migration [37]. The lack of wound closure in cells exposed to 7.5 and 10 mM MGO could significantly contributed by higher toxicity caused by excessive ROS production and DNA damage event exacerbated by decline in hTERT mRNA expression [38].

AGE are heterogenous compounds generated by spontaneous reactions via the addition of carbonyl group into free amino group of a protein, amino-phospholipid, or nucleic acid [11]. Although AGE accumulation occurs slowly, the deposition of these molecules could be elevated by high dietary consumption of glucose and fructose. The presence of excessive glucose stimulates methylglyoxal (MGO) production which was reported to be one of the most toxic AGE precursors on HaCaT cell line by causing reduced cell viability, interrupted cell cycle, and increased apoptosis due to elevated ROS level [35].

In this study, we quantified the concentration of ROS using H_2_DCFDA which could be converted to DCF fluorophore by the presence of hydroxyl, peroxyl, and other class of ROS intracellularly. Hydroxyl radical is known as the most reactive oxidant in the mitochondrial matrix, which may induce DNA damage by strand excision and oxidizes purine and pyrimidine bases [39]. In other hand, peroxyl radical causes DNA damage by attacking C-5 methylated at CpG sites in DNA and via lipid peroxidation induced genotoxicity [40]. DNA damage event in this study was visually revealed by running the extracted DNA in agarose electrophoresis. Our findings showed that there were increased in DNA smearing pattern in cells treated with 7.5 and 10 mM MGO. The presence of more visible DNA smearing might be partially promoted by the reduction of hTERT expression, which important in telomere length maintenance [38].

The toxic effect of MGO has been extensively studied mostly in diabetic settings, especially its involvement in inducing diabetic sensorimotor polyneuropathy (DSPN), neuropathic pain, endothelial dysfunction, and functional alterations of human insulin. To test for the mechanism for DSPN due to MGO, a study has shown that the exposure of 500 µM MGO led to reductions of undifferentiated SH-SY5Y neuroblastoma cells to 50 %. The same study also showed declines in neurite counts and its average lengths [41]. Interestingly, another study suggested that astrocytes, which are known to have higher glucose metabolism, were 6-folds more resistant to MGO which a significant viability reduction was reported after treated with 3.39 mM MGO [25]. Other reports also demonstrated higher concentrations of MGO (10 mM) was required to induced cellular toxicity in RINm5F which may indicate the presence of functional insulin production [24]. The same concentration also described to promote the activation of human TRPA1 in HEK293t cells, which reported to potentially trigger the stimulation of cutaneous nociceptors in diabetic patients [42].

Several studies have reported the role of AGE as the final product of MGO in mediating the emergence of skin ageing signs. A clinical study revealed a significant positive correlation of AGE concentration in dermis with melanin count, erythema, and trans-epidermal water loss [43]. Moreover, a negative correlation of AGE with skin hydration were also reported significantly in the same study. Another report also revealed increased concentration of AGE contributed to skin pigmentation by activating the expression of NLRP3 and IL-18 in sun-exposed skin samples. This evidence strongly implies that the impact of AGE during the manifestation of skin ageing [44].

Despite having demonstrated the cellular and molecular consequences of methylglyoxal in HDFa, there were several limitations to be mentioned. First, this study did not cover the potential programmed cell death mechanism to identify whether treatment of MGO on HDFa led to necrosis, apoptosis, or ferroptosis due to technical limitation. Moreover, the experimental replication on neonatal dermal fibroblast were not performed due to limitation in sample resources. Nevertheless, this study provided evidence regarding the role of RAGE and hTERT expression with the cellular mechanism of reduced survival and impaired migration due to MGO exposure as the potential inducers of dermal ageing.

## 5 Conclusion

To summarize, we examined the cellular toxicity of MGO in primary adult human fibroblast (HDFa). Our findings demonstrated the exposure of MGO on HDFa led to reduction in HDFa survival and migration, which we argue was partly due to oxidative stress condition triggered by the activation of receptor of advanced glycation end-products (RAGE). The activation of AGE-RAGE was previously reported to stimulate NOX generating enzyme and limit the antioxidant defence system. This situation poses a high risk of DNA damage event which could be exacerbated by reduction of hTERT expression in cells exposed to toxic doses of MGO in our report.

## Ethics approval

Ethical exemption letter (KET-451/UN2-F1/ETIK/PPM.00.02/2024) was given by The Health Research Ethics Committee, Faculty of Medicine, University of Indonesia-Cipto Mangungkusumo Hospital.

## Informed consent

Not applicable.

## Funding

The authors are grateful for the funding provided by The Indonesian Endowment Fund for Education (LPDP) organized by RIIM Grant of National Research and Innovation Agency (BRIN) with contract ID: 36/IV/KS/06/2022.

## Declaration of Competing Interest

The authors declare that they have no known competing financial interests or personal relationships that could have appeared to influence the work reported in this paper.

## Data Availability

Data will be made available on request.
